# Enzymatic Reduction of 9-Methoxytariacuripyrone by *Saccharomyces cerevisiae* and Its Antimycobacterial Activity

**DOI:** 10.3390/molecules17078464

**Published:** 2012-07-12

**Authors:** Patricia Alvarez-Fitz, Laura Alvarez, Silvia Marquina, Julieta Luna-Herrera, Víctor Manuel Navarro-García

**Affiliations:** 1 Laboratory of Microbiology, Biomedical Research Center of the South (IMSS), Argentina 1, Col. Centro, 62790 Xochitepec, Morelos, Mexico; 2 Center for Development of Biotic Products(IPN), Carretera Yautepec-Jojutla, Km. 6, Calle Ceprobi 8, Col San Isidro, Apdo. Postal 24, 62731 Yautepec, Morelos, Mexico; 3 Chemical Research Center, University of Morelos (UAEM), Av. Universidad 1001, Col. Chamilpa, 62209 Cuernavaca, Morelos, Mexico; 4 Immunochemistry Laboratory II, Department of Immunology, National School of Sciences Biology (IPN), Prolongación Carpio y Plan de Ayala S/N, 11340, D.F., Mexico

**Keywords:** biotransformation, *Saccharomyces cerevisiae*, 9-methoxytariacuripyrone, antituberculous activity

## Abstract

Biotransformation processes have been successfully utilized to obtain products of pharmaceutical, chemical, food, and agricultural interest, which are difficult to obtain by classic chemical methods. The compound with antituberculous activity, 9-methoxy-tariacuripyrone (**1**), isolated from *Aristolochia brevipes*, was submitted to biotransformation with the yeast *Saccharomyces cerevisiae* under culture, yielding 5-amino-9-methoxy-3,4-dihydro-2*H*-benzo[h]chromen-2-one (**2**). The structure of **2** was elucidated on the basis of spectroscopic analyses. The results mainly show the reduction of the double bond and the nitro group of compound **1**. Metabolite **2** demonstrated an increase in anti-tuberculous activity (MIC = 3.12 µg/mL) against the drug-sensitive *Mycobacterium tuberculosis* (H37Rv) strain, with respect to that shown by **1**.

## 1. Introduction

During the past decade, biotransformations have attracted increasing interest and currently comprise one of the most promising areas in research, due to their possible application in obtaining raw materials and useful products in different industrial processes and in important sectors such as pharmaceuticals, chemicals, foods, and agriculture. These processes have been utilized very successfully in obtaining novel molecules and in the modification of organic substrates, with the objective of increasing their commercial or scientific value. The principal advantages of their use are the following: resolution of racemates, selective conversion of functional groups between groups of similar reactivity, introduction of a chiral center, and the functionalization of non-active carbons, in addition to the preparation of optically active compounds with high stereoselectivity under environmentally gentle and friendly conditions [1,2,3,4]. Biotransformation reactions have been carried out by diverse microorganisms such as bacteria (*Pseudomonas*), fungi (*Aspergillus niger*, *Penicillium*, *Cladosporium*, *Botrytis cinerea*), and some algae [5].

One of the most frequently employed microorganisms in biotransformation processes is the yeast *Saccharomyces cerevisiae* (baker’s yeast, BY), which from remote times has been used in the production of alcoholic beverages and in the bakery industry. Edible and industrial ethanol production represents the majority of *S. cerevisiae* use in biotechnological applications. However, baker’s yeast also plays an important role as a model organism in the field of biochemistry, genetics and molecular biology [6].

It is well known that BY contains enzymes that carry out several chemical reactions, such as: asymmetrical reduction of the carbonyl group to an alcohol group with high yields; carbonyl compounds with several substituents (Me, Et, *n*-Pr, *n*-Bu, Bz) are also reduced [4], and some aldehydes, oxo esters, and α-oxo esters are also reduced by BY [7]. Thus, BY is widely utilized as a bio-reagent in chemical synthesis [8]. Moreover, BY promotes the reduction of nitroaromatic compounds to their respective amines utilizing very simple reactions and without producing any harmful effect [9].

We studied the plant species *Aristolochia brevipes* (*Aristolochiaceae*), which grows in several states of the Mexican Republic such as Michoacán, Colima, Guerrero, and Morelos, where it is popularly known as “guaco” and widely used in Mexican traditional medicine to treat arthritis, diarrhea, and snake-bite wounds. The plant’s rhizomes are the most utilized part [10,11,12]. Previous studies conducted on this plant species report the presence of 9-methoxytariacuripyrone (**1**) and 7,9-dimethoxy-tariacuripyrone [10], as well as aporfinic alkaloids such as 6a,7-dehydro-*N*-formylnornantenine, *N*-formylnornantenine, and aristololactame I [12]. Additionally, the anti-tuberculous activity of these compounds has been reported [13].

**Figure 1:**
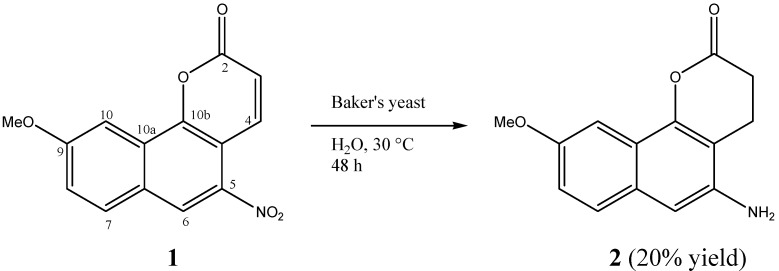
Biotransformation of **1** with *Saccharomices cerevisiae* in culture.

The objective of this work was to submit **1** to biotransformation with BY and to evaluate the antimycobacterial activity of its transformation products. The biotransformation of 9-methoxytariacuripyrone (**1**) with *Saccharomyces cerevisiae* afforded metabolite **2** (Figure 1). The present contribution describes the production, isolation, structure elucidation, and antimycobacterial activity of this metabolite.

## 2. Results and Discussion

**Figure 2:**
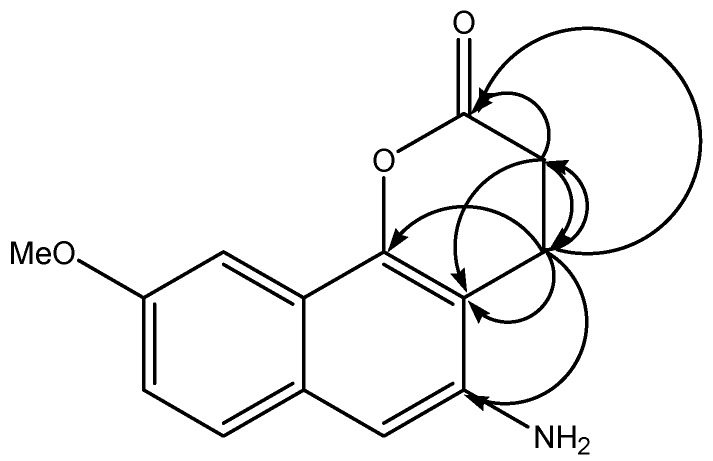
Selected HMBC correlations for compound **2**.

Compound **2** was obtained (20% yield) from the biotransformation of **1** with BY, and it displayed a quasi-molecular ion peak at *m/z* 266.2578 corresponding to the molecular formula C_14_H_13_NO_3_Na, indicating a metabolite structure with two less oxygen atoms than **1**. Its infrared (IR) spectrum demonstrated the disappearance of the nitro and double bond groups and the appearance of the NH_2_ absorption band at 3,416.9 cm^−1^, indicating that the reduction of these groups has occurred. The nuclear magnetic resonance (NMR) spectral data of **2** were similar to that of **1** with the exception of carbon and proton signals at positions 3, 4, 5, and 6. This observation was consistent with the absence of the α,β unsaturated hydrogens signals of H-3 and H-4 and the appearance in the ^1^H-NMR of two triplets at δ 3.14 (2H, t, *J* = 4.0 Hz) and δ 2.86 (2H, t, *J* = 4.0 Hz), as well as two aliphatic carbon signals at δ 21.33 (C-3) and 35.0 (C-4) which were each correlated in the HMQC spectrum. These signals were assigned to H-4 and H-3, respectively, by mean of the correlations observed between the signal at δ 3.14 with C-2, C-3, C-4a, -C-5, and C-10b and between the signal at δ 2.86 with C-2, C-4, and C-4a in the HMBC spectrum (Figure 2). The presence of a broad singlet signal at δ 5.02 characteristic of an amine group, as well as the upfield shift of the H-6 signal (Δδ = 0.56 ppm), and the downfield shift of the C-5 signal (Δδ = 3.1 ppm) in the ^13^C-NMR spectrum indicated the reduction of the nitro to the amine group [10]. Thus, the structure of this metabolite was determined as 5-amino-9-methoxy-3,4-dihydro-2*H*-benzo[h]chromen-2-one. This compound has not been isolated previously.

From the data obtained by NMR, we proved that BY performed the reduction of the nitro group to an amine; it is known that the reaction of this type of chemical group is due to nitroreductase enzymes [14,15].

Biodegradation of nitroaromatics can be initiated by both reductive and oxidative mechanisms. The reductive pathways involve conversion of the nitro substituent to an amino group through the action of nitroreductases [15,16,17]. Type I (oxygen-insensitive) nitroreductases catalyze the sequential transfer of two electrons from NAD(P)H to the nitro groups of nitrosubstituted compounds, in the presence or absence of oxygen, resulting in nitroso and hydroxylamine intermediates and finally primary amines [18,19,20]. Many nitroreductases have been reported to possess broad substrate specificity catalyzing the reduction of organic nitroaromatic and nitroheterocyclic compounds [17,21,22,23,24].

It was also determined that BY carried out the reduction of the double bond in positions 3 and 4 of the substrate **1**. BY-mediated double-bond reduction is known. For example, Woodward’s lactones are reduced in the double bond, with a yield of nearly 20%; likewise, the transformation of oxoisophorene (3,5,5-trimethyl-2-cyclohexene-1,4-dione) with BY produces four reaction products, of which two correspond to the double-bond reduction, this reaction being very fast and carried out at room temperature (30 °C) [4]. This is the first report of the reduction of **1** by the yeast *Saccharomyces cerevisiae*.

Compound **2** was finally evaluated with the Microplate Alamar Blue Assay (MABA) method to determine its antituberculous activity, and the result showed that this metabolite (MIC, 3.125 µg/mL) was more active than its parent compound **1** (MIC, 25 µg/mL, [13]) against *Mycobacterium tuberculosis* H37Rv.

## 3. Experimental

### 3.1. General

Melting points were determined on a Fisher Johns melting point apparatus. The IR spectra were obtained in KBr or as films (CHCl_3_) on a Bruker Vector 22 IR Spectrometer. CIMS spectra were recorded on a Jeol JMX-AX 505 HA mass spectrometer in a matrix of glycerol. Structure was determined through ^1^H and ^13^C nuclear magnetic resonance (NMR) experiments, which were obtained in CDCl_3_ on Varian Unity 400 equipment, at 400 MHz for ^1^H, and at 100 MHz for ^13^C utilizing tetramethylsilane (TMS) as internal reference. 

### 3.2. The Biotransformation Process

The procedure was performed according to that reported by Takeshita *et al.* [16], with some modifications for this study. We added distilled water (200 mL) and *Saccharomyces cerevisiae* yeast (50 g, NEVADA®, Edo. México, México) to 500 mL Erlenmeyer flasks; these were shaken at a temperature of 30 °C at 160 rpm for 1–2 h, the initial pH was 5.5. After this time, we added the substrate 9-methoxytariacuripyrone (**1**, 100 mg) dissolved in ethanol (1 mL) to the reaction flasks, while the control flasks only contained *Saccharomyces cerevisiae*. The flasks were again shaken under the aforementioned conditions during 48 h. The progress of the transformation reaction was monitored using thin layer chromatography (TLC), in which 10–15-mL aliquots were taken from the reaction mixture, extracted by partition with ethyl acetate (25 mL), and the organic phase was concentrated under reduced pressure for analysis utilizing 9-methoxytariacuripyrone (**1**) as reference. 

### 3.3. Extraction, Purification and Elucidation of Biotransformation Product

The mixture (pH was adjusted to 7.0 using 2M sodium hydroxide solution) was extracted with ethyl acetate (500 mL × 3 times). The organic phase was filtered through a Celite bed; afterward, the organic phase was concentrated at reduced pressure to obtain the extract that contains the reaction products.

For purification of the products, we employed open column chromatography (OCC), for which we utilized silica gel (Merck Kiesegel 60, Darmstadt, Germany). This was eluted with a 9:1 hexane-acetone system, gradually increasing the polarity with acetone. The fractions and/or products obtained were monitored using TLC and were observed with ultraviolet (UV) light and revealed with ammonium sulfate solution (Sigma Chemical Co., St. Louis, MO, USA); the fractions were again rechromatographed by means of OCC in order to obtain the pure products. 

*5-Amino-9-methoxy-3,4-dihydro-2H-benzo[h]chromen-2-one* (**2**). Yellow powder; m.p. 118–120 °C; HRFABMS (positive mode) *m/z*, calculated for C_14_H_13_NO_3_Na: 266.2513, found: 266.2578; IR: ν (cm^−1^): 3416.9, 1260 (NH_2_), 1627.05 (C=O); ^1^H-NMR: δ = 2.86 (t, *J* = 4 Hz, H-3), 3.14 (t, *J* = 4 Hz, H-4), 7.95 (s, H-6), 7.79 (d, *J* = 8.8 Hz, H-7), 7.19 (dd, *J* = 8.8, 2.8 Hz, H-8), 7.61 (d, *J* = 2.8 Hz, H-10), 3.96 (s, 9-OMe), 5.02 (s, NH_2_-5); ^13^C-NMR: δ 181.70 (C-2), 21.33 (C-3), 35.0 (C-4), 117.97 (C-4a), 149.45 (C-5), 118.15 (C-6), 131.58 (C-6a), 132.3 (C-7), 122.25 (C-8), 162.06 (C-9), 102.91 (C-10), 128.58 (C-10a), 152.5 (C-10b).

### 3.4. Antimycobacterial Assay

The antimycobacterial activity was determined by the fluorometric Microplate-based Alamar blue assay (MABA) method. The methodology was fully described by Luna-Herrera and Navarro-García *et al.* [13,25].

## 4. Conclusions

The results indicate that BY possesses the enzymatic capacity to reduce the nitro group in position C-5 of compound **1** to an amine group, as well as to reduce the double bond in positions of C-3 and C-4. In the yeast *Saccharomyces cerevisiae*, two nitroreductase proteins, involved in the oxidative stress response, Frm2p and Hbn1p, have been described, [18,26], and could be the responsible of the reduction reactions observed in this biotransformation of **1**.

The reduction of the nitro and double bond groups resulted in an increase in antituberculous activity tested against *Mycobacterium tuberculosis* H37Rv. With the results found here, we provide the opportunity to conduct new pharmacological and chemical studies on this substrate. This type of reaction can be useful in obtaining new products, which could increase the therapeutic arsenal against a disease that is exhibiting resurgence with resistant bacteria, such as is the case of tuberculosis.
